# A late Pleistocene human footprint from the Pilauco archaeological site, northern Patagonia, Chile

**DOI:** 10.1371/journal.pone.0213572

**Published:** 2019-04-24

**Authors:** Karen Moreno, Juan Enrique Bostelmann, Cintia Macías, Ximena Navarro-Harris, Ricardo De Pol-Holz, Mario Pino

**Affiliations:** 1 Instituto de Ciencias de la Tierra, Facultad de Ciencias, Universidad Austral de Chile, Valdivia, Chile; 2 Transdisciplinary Center for Quaternary research in the South of Chile, TAQUACh, Valdivia, Chile; 3 Departamento de Antropología, Universidad Católica de Temuco, Temuco, Chile; 4 Centro de Investigación Gaia Antártica (CIGA) and Center for Climate and Resilience Research (CR)^2^, Universidad de Magallanes, Punta Arenas, Chile; National Cheng Kung University, TAIWAN

## Abstract

The present study describes the discovery of a singular sedimentary structure corresponding to an ichnite that was excavated at the paleo-archaeological site Pilauco (Osorno, Chile). The trace fossil is associated with megafauna bones, plant material and unifacial lithic tools. Here we present a detailed analysis of the Pilauco ichnite and associated sedimentary structures, as well as new radiocarbon data. The ichnological analysis confidently assigns the trace to the ichnospecies *Hominipes modernus*—a hominoid footprint usually related to *Homo sapiens*. Some particular characteristics of the Pilauco trace include an elongated distal hallux, lateral digit impressions obliterated by the collapsed sediment, and sediment lumps inside and around the trace. In order to evaluate the origin of the ichnite, trackmaking experiments are performed on re-hydrated fossil bed sediments. The results demonstrate that a human agent could easily generate a footprint morphology equivalent to the sedimentary structure when walking on a saturated substrate. Based on the evidence, we conclude that the trackmaker might well have been a bare-footed adult human. This finding, along with the presence of lithic artifacts in the same sedimentary levels, might represent further evidence for a pre-Clovis South American colonization of northern Patagonia, as originally proposed for the nearby Monte Verde site.

## Introduction

**Fig 1:**
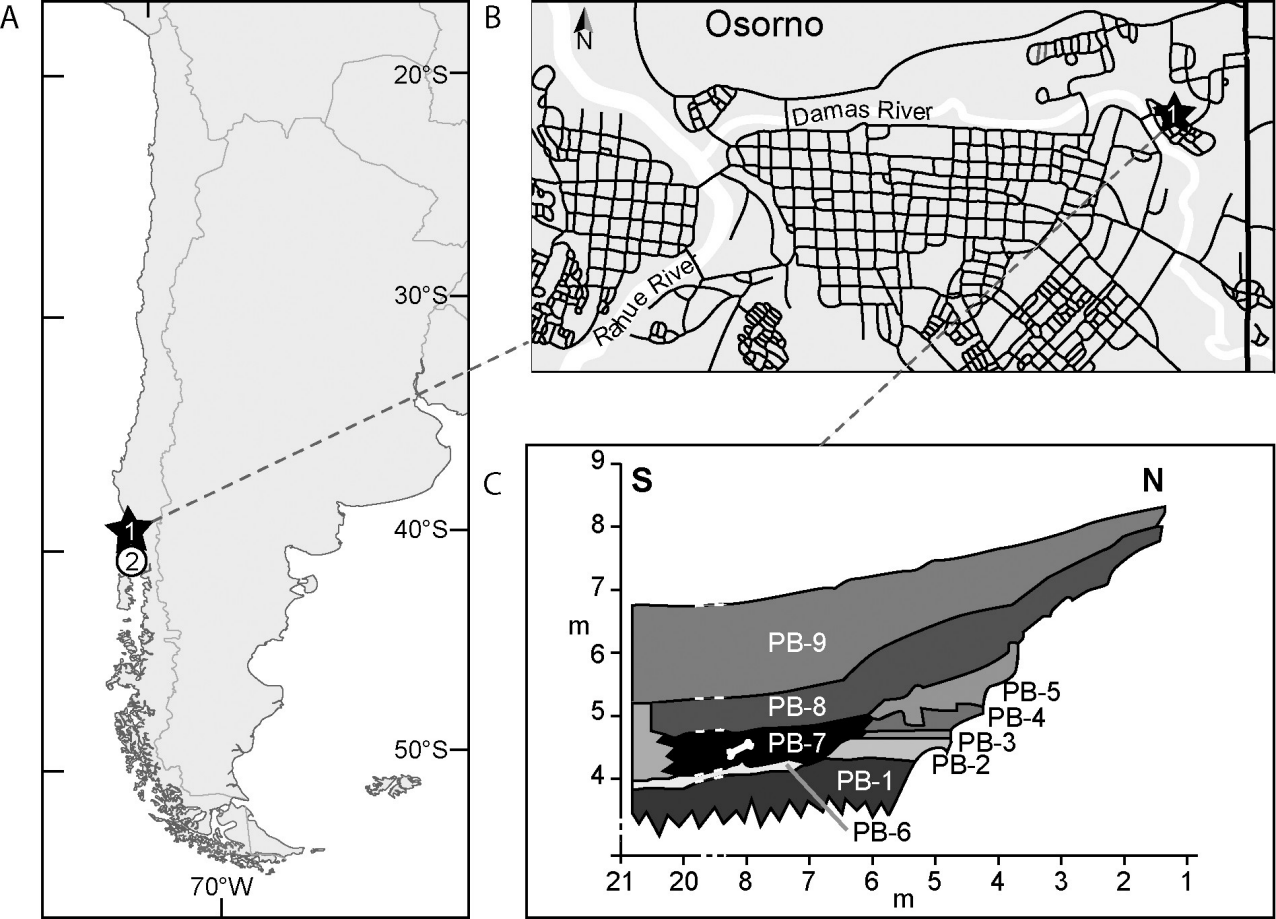
**A) Location of archeo-palaeontological sites in southern South America dated around 12.500** ^**14**^**C yr BP, including the Pilauco site (black star)**: 1) Pilauco, Osorno, Chile 2) Monte Verde, Chile. B) Detailed location of the Pilauco site next to the actual Damas River, at Villa Los Notros, Pilauco, street address: Río Cachapoal #159, Osorno. C) Schematic stratigraphy of the Pilauco site (modified from Pino et al., 2013). PB-1: Lapilli tuff; PB-2: Lapilli tuff with abundant volcanic ash matrix; PB-3: Lapilli tuff with medium sand matrix; PB-4: Volcanic ash; PB-5: Terrigenous coarse sand with scattered angular pebbles; PB-6: Gravel composed by clasts ranging from 1 to 15 cm in diameter; PB-7: Highly fossiliferous peat (indicated by a white bone drawing) with very dark brown matrix (10Y 3/1) including isolated and poorly selected gravel clasts up to 7 cm of Andean source. This layer is overlaying PB-2 and PB-6 by erosive unconformities; PB-8: Sandy peat (2.5Y 4/2), slightly more brown in color than PB-7. PB-9: Non-fossiliferous black peat (2.5Y 2/0). Both, PB-7 and PB-8 share a lateral facies change toward the South with an interfingering of gravel and sand layers.

While still controversial, late Pleistocene evidence of the peopling of South America is gaining more acceptance based on renewed interdisciplinary research on classic and recently discovered archeo-palaeontological sites [1–4]. The Monte Verde site is located in Chilean northwestern Patagonia and dated between 12,780 ± 240 and 12,230 ± 140 ^14^C year Before Present (yr BP) ~14,600 calibrated (cal) yr BP; [5,6]). It is certainly the best-known and mostly recognized site recording the early chronology of human presence in the subcontinent [2,7,8]. The Pilauco site is another late Pleistocene archeo-paleontological site that has been excavated and analyzed from 2007 in the city of Osorno (40°34’S– 73°07’W, Fig 1). The site is contemporaneous to Monte Verde and located 100 km from it. So far, the Pilauco site has provided a variety of evidence on late Pleistocene human, floral and faunal coexistence in northwestern Patagonia [9–15] (S1 File). The fossil remains collected at Pilauco during the 2007–2016 excavation seasons include large bones of fossil mammals, invertebrates, traces, wood fragments, seeds, vegetal materials, and a lithic assemblage composed of unifacial artifacts, flakes and debitage made from aphanitic and vitreous volcanic materials (S1 File).

**Fig 2:**
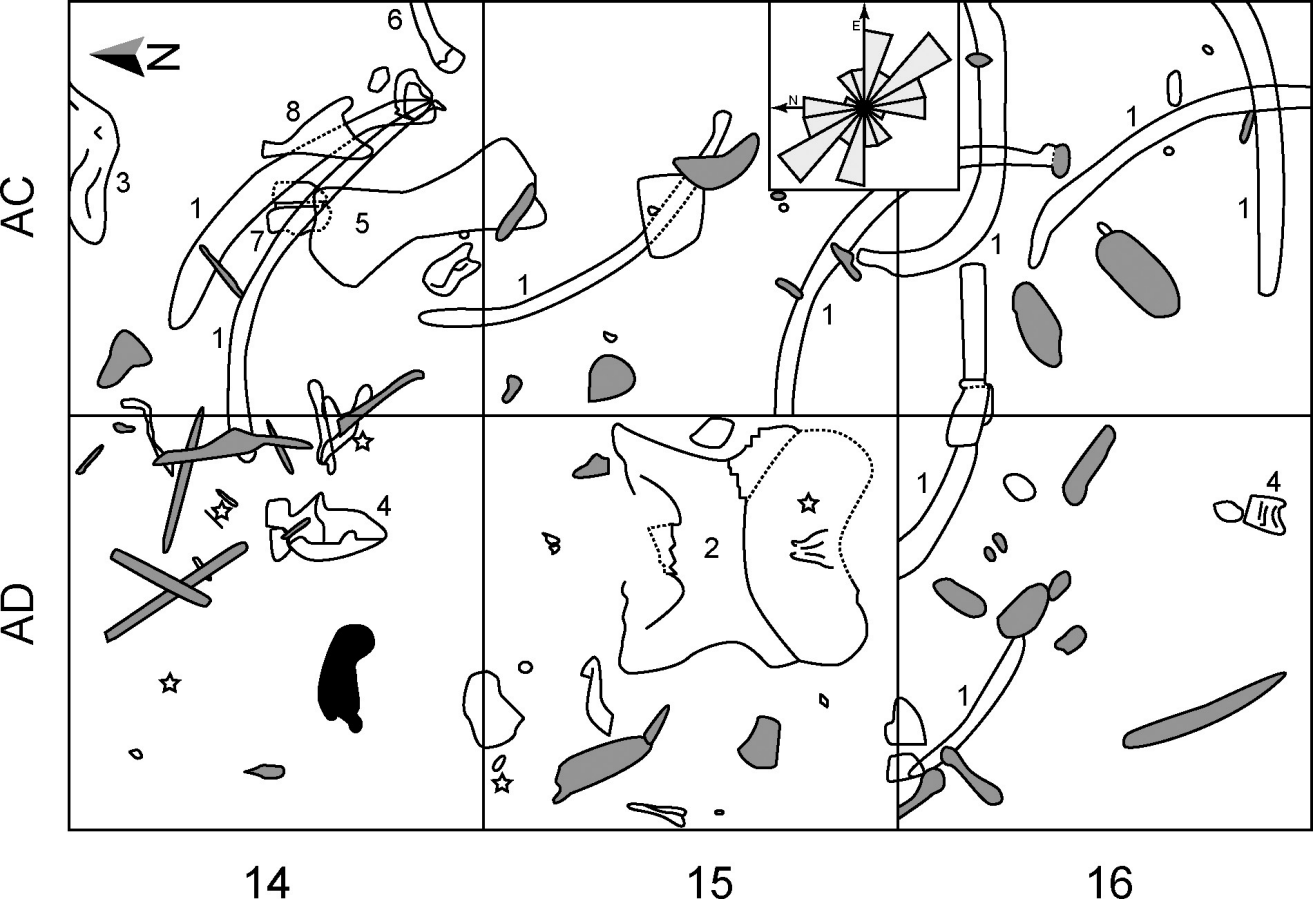
Schematic view of the PB-7 west excavation grid (only season 2011). Large pieces of wood (grey) and diverse bones can be observed. The sedimentary structure (“human paleoichnite”) described here (black) is in 14AD grid. *Notiomastodon platensis*: 1) ribs; 2) skull; 3) atlas; 4) dorsal vertebra; 5) tibia; 6) fibula; 7) astragalus; and 8) scapula of a smaller herbivorous mammal, equidae or camelidae. Notices that the four identified lithic tools (stars) are distributed on 14–15 AD grids only. Rose diagram insert indicates the preferential SE-NW direction of the fossil material.

The first extinct megafauna bones were recovered from Pilauco in 1986 during the construction of the Villa Los Notros in the Pilauco neighbourghood. The fossils were identified as mastodon and horse remains [15, 16] and the site remained intact until 2007. During the 2011 fieldwork, an ichnological structure was excavated from a single sandy-peat layer (PB-7), which corresponds to a swamp-like deposit on an ancient colluvial plain of the Damas River (Figs 1 and 2; S1 File). Besides the archaeological artifacts collected at Pilauco [17](S1 File), the presence of a human trace provides extraordinary independent evidence on the occurrence of late Pleistocene settlers in this stratigraphic context.

In the present study, we provide a detailed description of this sedimentary structure and offer an ichnotaxobase based on modern ichnological criteria. Sedimentological and stratigraphic analyzes, as well as new radiocarbon data are also presented. The experimental approach implies a test for different scenarios aiming at the original conditions for the formation of the footprint, discussing its sedimentological characteristics, and the nature of the track-making agent.

## Material and methods

No human or animal research was involved in the present study. Site excavation was officially permitted by the The National Monuments Council, Chile.

### Fossil materials

The human footprint was found within the 14AD grid, near the base of the PB-7 basal layer, where abundant fossil bones of megafauna and plant materials have been found (Figs 1 and 2, S1 File). The structure was originally recognized due to a noticeable change in consistency between the ichnite infilling (which was easily removed) and the more compressed, firm stratum, that gave shape to the sedimentary structure (“fossil trackbed”).

### Sedimentology and stratigraphy

Sediment samples were taken from grids 14AD, 14AC, 15AD and 15AC as well as from the footprint infilling material. The sedimentary composition of these samples was analyzed by standard methods using wet sieving and an anti-flocculent agent to separate the mud, sand and gravel fractions [18]. The organic content of the samples was estimated by the method of ignition [19].

Wood and other vegetal remains picked from sediments of the 14AD grid and the infilling material were used for radiocarbon dating. Analyzes were carried out at the Keck Carbon Cycle Radiocarbon Laboratory of the University of California at Irvine. Calibrated dates were obtained with Calib 7.1, using the SHcal13 calibration curve [20] (accessed through http://calib.org).

### Ichnological analysis

**Fig 3:**
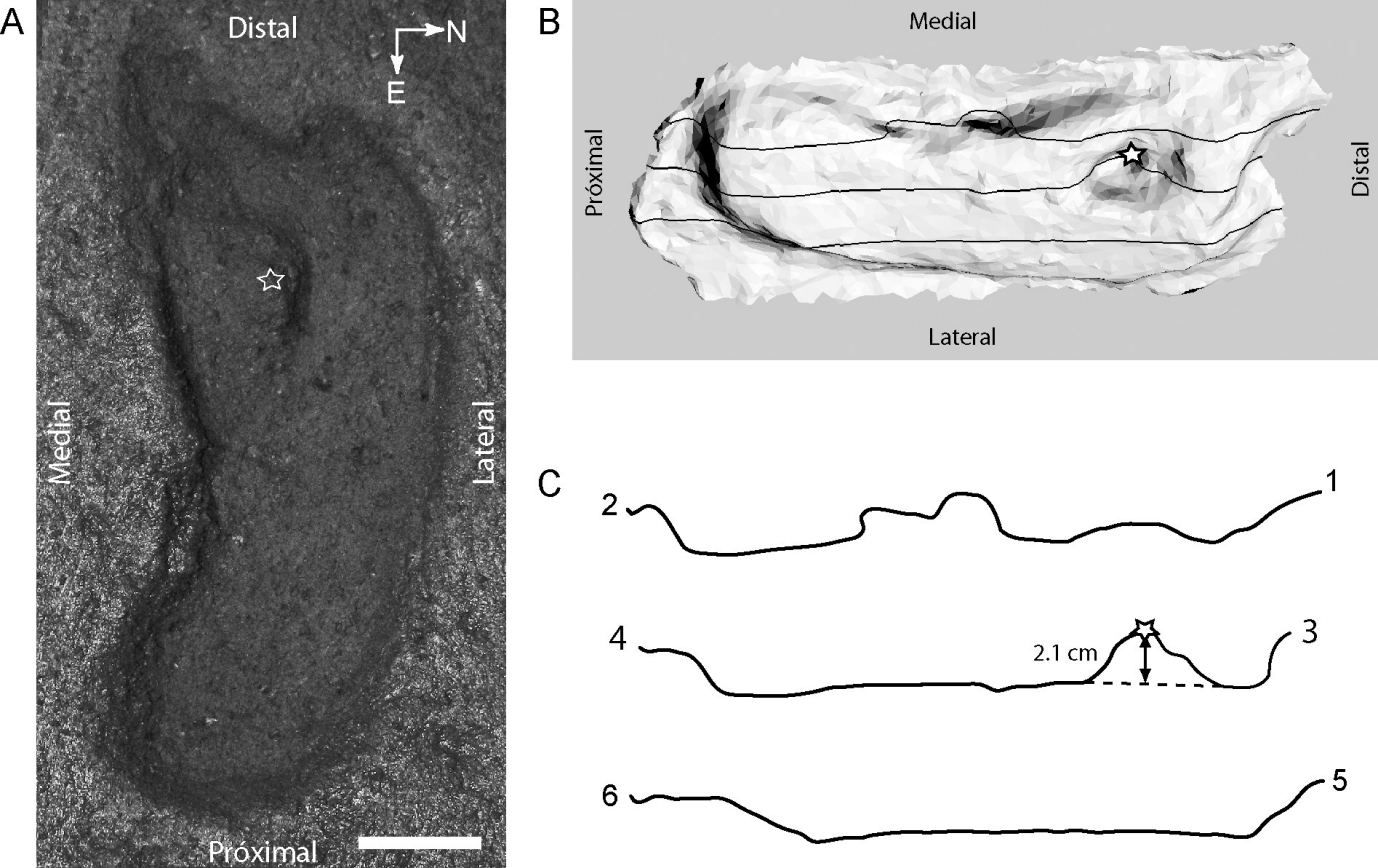
**A) Photography of the original sedimentary structure attributed to a human footprint that was excavated at the Pilauco site. A sediment lump is apparently embedded within the trackbed (star). Scale bar 5 cm.** B) Three-dimensional model in dorsal view with a virtual 45° tilt toward the south to facilitate the observation of profile lines 1–2, 3–4 and 5–6 drawn on the 3D model surface (123Catch from Autodesk and trial version of Rhino4, McNeel &Associates). C) Profile lines: [1–2] crossing from the “heel”, “medial longitudinal arch” and “hallux”; [3–4] passing by the midline. Notice that the sediment lump is 2.1 cm high from the footprint base; and [5–6] line passing through the “heel”, “lateral longitudinal arch” and “lateral digits”.

The *in situ* measurements of the ichnite were performed with common measuring tape and a metallic ruler. A commercial silicone cast was made, and X-ray images were taken using a General Electric DXD 350 radiographic equipment, with a CR Vita k401186 digitalization unit (Fig 3). Trace characters were observed and codified following Lockley et al. 2007–9, Kim et al 2008 [21–24], and compared to other potential non-anthropogenic ichnotaxa of similar dimensions such as ground sloths [25, 26] (S1 File). The sediment block containing the original structure was extracted from the field, placed in a glass box for conservation under the collection number MHMOAR/PI/55, and is housed at the recently established Pleistocene Museum in the Parque Chuyaca, Osorno, Chile.

### Experimental design

A virtual three-dimensional mesh was constructed using a series of pictures taken from different angles of the silicone cast and processed using the software 123D Catch (free license, 2013 Autodesk, Inc.). Generated files were coded with “obj” formats and analyzed with the 3D design software Rhinoceros (trial version of Rhino4, McNeel & Associates; Fig 3). The virtual 3D mesh is available from doi:10.5061/dryad.fh576k0.

A set of nine experiments was performed to test for different scenarios regarding the footprint formation, using three different water contents on sediments, and three different human barefoot trackmakers with similar foot size, but different height and weight (S1 File). A 230 cm long, 80 cm wide and 10 cm deep box was constructed and placed on a flat surface under shadow to avoid desiccation. It was filled up with ~0.18 m^3^ of sediments extracted directly from the layer near the excavated ichnite on grid 14AD. Trackmakers walked on the sediment immediately after setting up the box, with a maximum of a 15-minutes pause in between for: 1) taking pictures with scale, 2) measuring the hallux, heel and medial longitudinal arch depths of each footprint with a measuring tape and a ruler serving as a surface level, and 3) flattening of the trackbed surface. Subsequent tests were made after mixing 12 and 38 l of additional water, respectively.

Before each experimental trackway, sediment samples were analyzed for their water content. Data on sample weight before and after total desiccation after 48 hrs. at 40°C, indicated 36%, 44% and 68% water content on each sediment trial, which were coded as “dry”, “wet” and “saturated” water content respectively.

Walking speed was estimated using Alexander’s formula noted as: Speed = 0.25 g^0.5^ x SL^1.67^x H^-1.17^, where g is the gravitational acceleration (9.81 m/s^2^), SL is the stride length (distance between two footprints of the same foot) and H the hip height [27]. The formula has proven useful and accurate for a wide variety of animals, including humans, since it is based on the geometrical similarity between terrestrial vertebrates [28–32]. The final data were used for relative comparison of the walking effort.

## Results

### Sedimentology and stratigraphy

Textural data indicate that the trackbed’s gravel and mud content appears to be slightly higher than the infilling and adjacent sediments. The infilling and surface sediments show a slight increase in the organic component (11.1 and 10.5 wt% of organic matter, compared to 9.3 wt% for remaining samples, but generally these variations are minor (9.7 ± 1.4 wt%).

Radiocarbon dating of seeds and wood let to mostly consistently younger ages toward the top of the stratigraphic profile (Table 1), reflecting a low energy setting with only mild perturbations. For the underlying strata PB-6 the mean of the two median probability ages is 17,300 cal yr BP (Table 1). The age of the footprint is constrained by seven ages ranging between 13,195 ± 35 and 12,735 ± 40 14C yr BP. Thus, according to the median probability ages, it is safe to say that the Pilauco footprint is *circa* 15,600 cal yr BP. Additional radiocarbon dating obtained from a portion of a *Notiomastodon platensis* skull found near the trace, as well as a sample taken from a rib and its infilling sediments, indicate ages of 13,220 ± 60 and 13,240 ± 60–12,905 ± 40 ^14^C yr BP, respectively [10]. It is completely normal to find these age ranges, since wood, seeds, and/or bone material have separate depositional histories within the site, and these with the footprint itself, which cannot be directly dated.

**Table 1:** Radiocarbon dates and probable calibrated median age of various samples of plant material from under, within, on, and below the footprint.

#	Lab code	Description	d^13^C (‰ PDB)	^14^C age	Median Probability	2d range
1	101672	piece of wood from above the trackbed’s infilling (PB7)		12,860 ± 35	15,270	15,471–15,112
2	101770	seed from the trackbed’s infilling (lab. duplicate of 101671)	-27.7	13,045 ± 30	15,560	15,332–15,749
3	101671	seed from the trackbed’s infilling (lab. duplicate of 101770)	-26.8	13,470 ± 35	16,156	15,977–16,320
4	101771	seed from below the trackbed’s surface (lab duplicate of 101673)	-28.0	12,735 ± 40	15,123	14,892–15,289
5	101673	seed from below the trackbed’s surface (lab duplicate of 101771)	-27.7	13,145 ± 35	15,727	15,505–15,945
6	101769	seed from below the trackbed’s surface	-29.4	13,175 ± 40	15,770	15,987–15,573
7	101674	piece of wood from below the trackbed’s surface		13,195 ± 35	15,800	16,000–15,627
8	101675	piece of wood from the top of PB-6		14,195 ± 35	17,230	17,441–17,042
9	101676	piece of wood from the top of PB-6		14,300 ± 40	17,370	17,543–17,161

Dates were taken at the Keck Carbon Cycle Accelerator, Mass Spectrometry Laboratory Irvine California (UCIAMS). Calibration was achieved with CALIB 7.1, curve SHcal13 (Stuiver y Reimer, 1993; Hogg *et al*. 2003, http://calib.org consulted on February 2018).

### Systematic palaeoichnology of the pilauco trace

Ichnofamily Hominipodidae Kim et al. 2008

Ichnogenus Hominipes Kim et al. 2008

*Hominipes modernus* Kim et al. 2008[22]

Holotype: CU 230.135 plastic replica of a left foot impression, part of a trackway deposited in the Museo Municipal Huellas de Ancahualinca, Managua, Nicaragua [21,23].

Diagnosis: solitary or aggregated footprint impressions, plantigrade, with elongated axial plane, entaxonic, generally pentadactyle; with short, round, oval to slightly elongated digit prints, which may or may not be present because of taphonomic conditions. Print of digit I oval and extended in antero-posterior direction, and twice as long as the remaining digits. Anterior third region wider than the rest of the trace, and separated from the impressions of the digits by an axial ridge. Medial arch well demarcated and concave with respect to the inner edge of the impression (S6 Fig). Sub-circular heel mark (ball) projected internally, and narrower than the anterior third. Traces of cosmopolitan distribution, recorded from the early Pleistocene to the late Holocene, and assigned to bipedal hominids of the genus *Homo*, mainly *Homo sapiens*.

In America, Mexican 40,000 years old footprints, though contested [33], are of a similar size than the Pilauco footprint (26–27) cm long [34,35]. Also Pehuen Co’s human footprint sizes varies within the range (23–35 cm long) [36,37].

Due to its dimensions, general shape and taphonomic attributes, the ichnite of Pilauco corresponds to a right foot impression of an adult human, and discard other produces such as ground sloths (S1 File). The general length and width, together with the presence of a planar surface with a well-defined medial arch and a rounded posterior heel, allow to identify this trace as *Hominipes modernus* [10, 22]. The print has an elongated and semi-oval shape, with internally curved longitudinal axis, forming a left-projected longitudinal arcade that extends from the medial arch to the ball (Fig 3). The total linear length measured in the silicone replica from the anterior edge of digit I impression to the back of the heel is 279 mm, while the largest width, located in the mesial portion of the anterior third, (i.e. the “ball” of the foot), is 105 mm. The smaller width, at the height of the medial constriction, which is maintained toward the back of what could be interpreted as the heel, is 82 mm (Fig 3). The estimated FI footprint index is 0.38, placing the Pilauco footprint within the range of the larger and more robust individuals described for the ichnospecies [22,38](S1 File).

The plantar surface impression is flat, undifferentiated, and lacks ridges and/or separations between the anterior third, the plantar vault, the plantar isthmus and the anterior portion of the ball. The outer edge of the footprint impression is practically straight along its entire length, and slightly curved at the height of the outer anterior portion of the heel. The medial arch is well defined, circumscribing a pronounced concavity in the inner margin. The ball has a sub-rounded back edge that is internally directed. In general appearance, the plantar surface of the footprint is equivalent to a neutral to slightly pronator type. The region of the anterior third is the widest, which is a typical feature of *Hominipes modernus*. The anterior edge presents a triangular invagination at the height of the IV digit. The distal end generates a small angle with respect to the external border, which is common in the impressions of this ichnospecies [22].

Of the five digits, only the impression of digit I is properly delimited. It has a concave sub-circular section with the longitudinal axis obliquely oriented with a 25° torsion angle with respect to the plantar surface. A crest between the base of digit I and the edge of the anterior third can not be observed. On the contrary, an isthmus somewhat narrower than the diameter of digit I connects both regions. Adjacent to the change in angle, the base of digit I is slightly elevated above the surface plane. The remaining digits (II to V) are not present or did not leave evident impressions. An oval projection, located in the middle portion of the anterior region of the trace, could eventually correspond to the mark produced by the IV and/or V digits. One of the notable features of the trace is an ellipsoidal, conical elevation or promontory located in the center of the anterior third (Fig 3).

### Experimental trackmaking

The estimated speed calculated from the footprint data is similar for all trackmakers in dry and wet substrate, despite differences in their physique. However, the trackmakers walking on a saturated substrate show a slight decrease in their speed associated with an increase in the footprint depth (Table 2).

**Table 2:** General characteristics of the volunteer trackmakers, and detailed footprint depth measured on the substrate at different water contents after experimental trackmaking.

Water content	Track-maker	Body weight (kg)	Standing height (m)	Hip height (m)	Foot length (cm)	Estimated speed (km/h)	Foot-print number*	Foot-print length (cm)	Footprint depth at the:		
									thumb (cm)	medial arch (cm)	heel (cm)
**LOW**	A	77	1.70	0.86	26.5	3.9	1	26.5	0.6	0.0	0.5
							2	26.4	0.4	0.0	0.3
	B	72	1.82	1.20	27.0	3.7	1	27.0	0.4	0.0	0.3
							2	27.2	0.2	0.0	0.1
	C	62	1.75	0.87	27.0	**	**	27.0	**	**	**
**MEDIUM**	A	77	1.70	0.86	26.5	3.9	1	26.4	5.4	3.8	4.5
							2	26.5	7.7	6.0	7.0
							3	26.5	6.0	4.0	5.5
	B	72	1.82	1.20	27.0	3.7	1	27.1	4.0	2.0	3.5
							2	27.0	4.5	2.0	3.5
							3	27.0	4.5	3.5	4.0
	C	62	1.75	0.87	27.0	3.9	1	26.5	3.6	2.0	3.0
							2	26.8	4.4	3.0	4.0
							3	27.0	5.0	3.0	4.5
**HIGH**	A	77	1.70	0.86	26.5	3.4	1	26.3	8.5	7.0	8.0
							2	26.4	8.0	6.5	7.0
	B	72	1.82	1.20	27.0	3.2	1	26.9	7.6	6.5	7.0
							2	26.8	7.5	6.0	7.0
							3	27.1	9.0	7.5	8.5
	C	62	1.75	0.87	27.0	3.3	1	26.4	8.5	7.0	8.0
							2	26.6	***	6.0	6.5

*Right-foot-impressions-count only

**The trackmaker did not leave impressions

***Mud collapse did not allow for thumb depth measurements

Trackmaker B is on grey background in all the experiments to facilitate visual data contrast.

**Fig 4:**
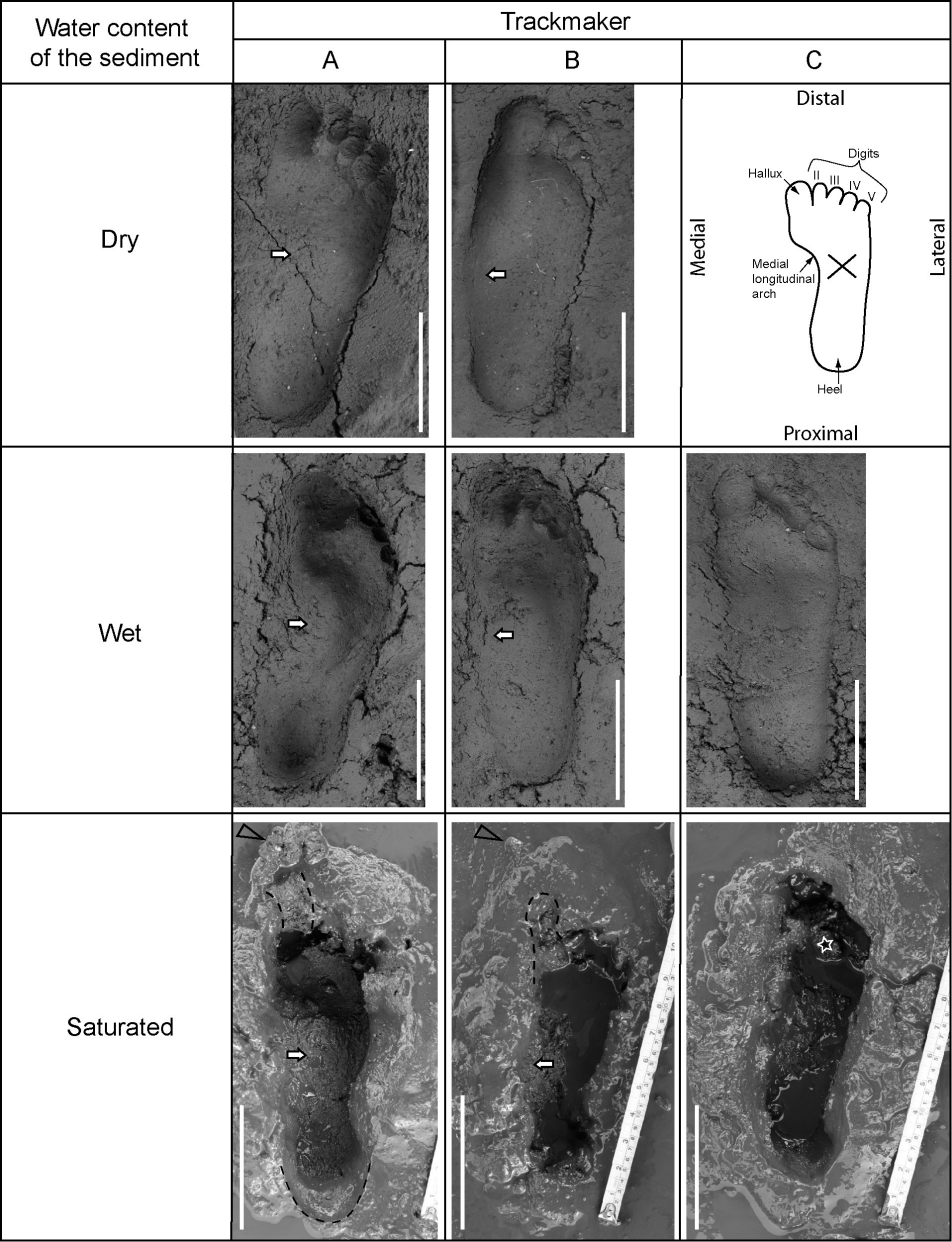
Experimental footprints. Each row corresponds to a trackmaker and each column to a dry, wet and saturated water content of the sediment, respectively. Notice that in all the experiments trackmaker A has a deeper forward imprint of the digits and a large medial longitudinal arch (white arrow), trackmaker B has a slightly more even distribution of the footprint’s depth, but shows no major signs of the medial longitudinal arch (white arrow), and trackmaker C leaves footprints only on wet and saturated water content substrates (concepts used here are schematized as a replacement of the absent dry water content imprint). This can be mostly explained by light body weight (at least 10 kg lighter than the other trackmakers), as well as a well-balanced center of gravity (trackmakers A and B have centers of gravity forwardly deviated). In general, the hallux is a prominent structure that can be identified on each experiment, while the imprint of the lateral digits is variable. On the saturated water content experiments the hallux can drag sediment forward (pointing triangle) or backward (star). Trackmaker C on saturated water content substrate left footprints remarkably similar to the sedimentary structure excavated in Pilauco.

The morphology of the experimental footprints varies along with the water content of the substrate (Fig 4). The measured lengths and footprint morphology throughout each trackway were consistent. Hence, only a single print of each trackway experiment is described.

On the dry substrate (Fig 4), trackmaker A presents deeper distal impressions consistent with higher body weight (Table 2) and a remarkable development of the medial longitudinal arch compared to the other trackmakers. Lateral digits drag the sediment backward and no displacement rims are observed. The hallux impression is well rounded. Trackmaker B shows a more uniform distribution of the footprint depth compared to trackmaker A. The medial longitudinal arch is rather absent and replaced by a wider medial border, related to a flat-footed condition. Lateral digit prints are easily distinguishable, but they slide slightly laterally producing a square-like shape. The hallux impression has a clear, well-rounded shape. No displacement rims are observed. Trackmaker C did not leave any footprint marks on the dry substrate setting. While substrate desiccation might have contributed to this result, it is important to note that trackmaker C has a noticeably lighter body weight (10–14 kg less than trackmaker A and B respectively, Table 2). His center of gravity appears to be better balanced than trackmakers A and B, which are forward deviated [34]. These factors might have a more significant effect than the eventual substrate desiccation. No displacement rims are observed.

On wet substrate (Fig 4), trackmaker A left deeper footprints compared to the other trackmakers. The medial longitudinal arch is well-developed. No displacement rims are observed. All digits sunk forward into the substrate, but are easily distinguishable showing a well-rounded shape. Trackmaker B left more uniformly distributed footprint-depth compared to trackmaker A. The medial longitudinal arch is rather absent and replaced by a wider medial border. The hallux impression is well-rounded, but lateral digits lose definition, and only digits II to IV are easily identifiable. No displacement rims can be observed. Trackmaker C left a generally shallow footprint, with similar proximal and distal depths. The medial longitudinal arch is slightly marked. The impression of the hallux and lateral digits are easily distinguishable and have well-rounded shapes. The hallux and the rest of the lateral digits compress digit II. No displacement rims are observed.

**Fig 5:**
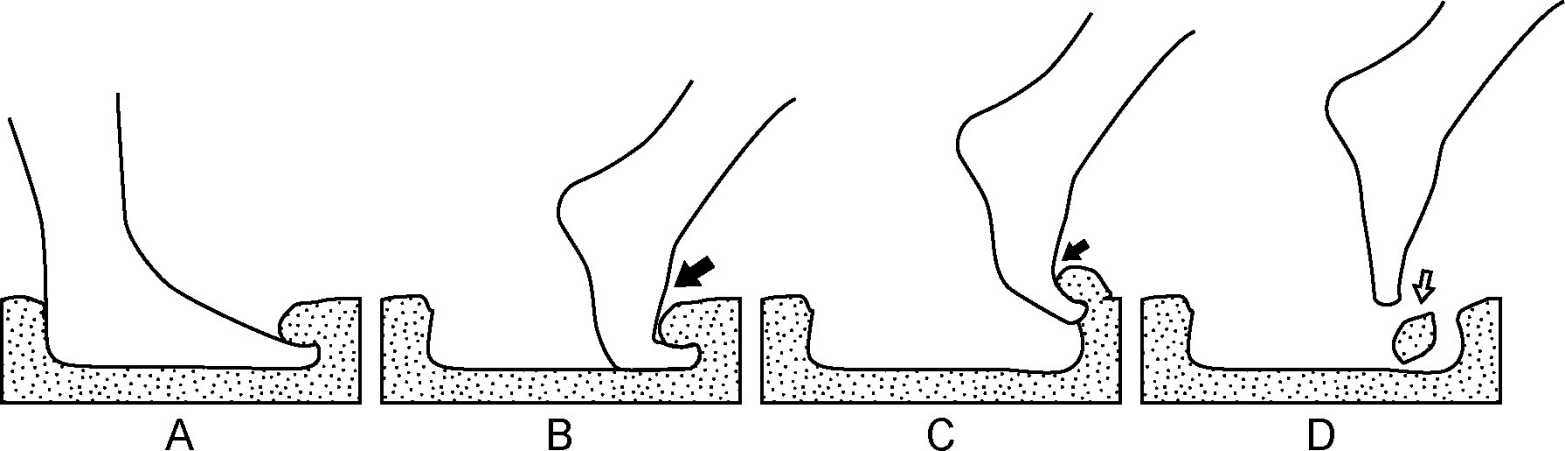
Schematic representation of pedal kinematics for trackmaker C explaining the mechanism of footprint formation on a substrate with saturated water content. A) standing phase, body weight deforms the substrate generating a displacement rim and a distal sediment collapse covers the dorsal surface of the toes (particularly on the hallux); B) initial lift-off phase, the trackmakers encounter a reaction force from the distal substrate decelerating progression (black arrow); C) nearly completed lift-off phase, only the digits are in contact with the distal substrate dragging a portion up and forward, a smaller reaction force counteracts foot movement (small black arrow); D) full lift-off phase, the substrate portion loaded on the toes drops down (white arrow) into the first distal half of the footprint.

Finally, in the saturated substrate (Fig 4), trackmaker A sunk a foot forward, producing a sediment wall through which the hallux passed dragging. This movement displaced some of the sediment forward, the hallux acting as a shovel carrying material and depositing it toward the distal surface of the impression (pointing triangle on Fig 4). The lateral digits are dragged into the distal surface, but the effect was less notorious and digits cannot be identified as separated structures. A marked displacement rim was observed all around the impression, becoming larger on the distal surface. Trackmaker B buried the pedal digits into the sediment. When lifting the foot up and forward it dragged the sediment forward, leaving the mark of each digit on the distal sediment wall. However, the hallux was dragging even further than the lateral digits. Some of the sediment trapped on the hallux’s dorsal surface dropped in front of the footprint (pointing triangle on Fig 4). Once the foot was lifted up, the sediment collapsed inside the imprint, filling up the hallux and digit II impressions. A marked displacement rim was observed all around the mark and is particularly notorious on its medial border. Trackmaker C left an elongated hallux mark, but the lateral digits are not identifiable. There is a sediment lump right behind the place where the digits should have left their mark. This lump dropped from the dorsal surface of the digits when the foot was lifted up and set forward (star on Figs 4 and 5). A marked displacement rim can be observed on the medial, proximal, lateral and latero-distal borders of the trace, but is absent in front of the hallux.

## Discussion

During the last 20 years, vertebrate palaeoichnology has experienced significant advancements in the constitution of ichnotaxobases, morphological characterizations, 3D reconstructions, identification of producers, and temporal and paleoenvironmental interpretations [24,25,39–46]. The study of hominid footprints and trackways in particular, identifies three monospecific currently valid ichnogenera: *Anthropoidipes ameriborialis* [39]; *Hominipes modernus* [21,24,38,45] and *Praehominipes laetoliensis* [42].

*Anthropoidipes ameriborialis* is easily distinguished from the Pilauco specimen because of its larger dimensions and robustness, the absence of a medial arch (sub-rectangular lateral borders), and the presence of an elongated heel region, aligned in an anterior-posterior direction. These characteristics also distinguish the Pilauco ichnofossil from the ichnogenus *Praehominipes laetoliensis* [24,42] which is characterized by sub-parallel trace edges with a fairly similar width-ratio between the anterior third and the ball. Consistent with these two ichnospecies, the footprint of Pilauco lacks differentiated structures in the plantar surface (i.e. an elliptical anterior third, a well-defined isthmus and anterior region of the rounded heel, and well delimited axial ridges). Nevertheless, several examples of *Hominipes modernus* lack these attributes as well, which can be directly related to the substrate consistency or later taphonomic biases [22].

The absence of a trackway prevents the identification of other important diagnostic features related to the divarication of the footprint angle. We do not know for sure why there has been a single footprint found at the site, but mild sediment mixing, and lateral fascies variation is a highly probable reason for differential preservational conditions. However, independent evidence of the archaeological record present at the site (S1 File) indicates *Homo sapiens* as the natural producer of the footprint. The record of the occurrence of *Hominipes* in South America so far has been restricted to the localities of La Olla, Pehuen Co and Monte Hermoso [36–47], along the Atlantic coast of Buenos Aires Province, Argentina. The last two sites include several dozens of specimens associated with a rich diversity of vertebrate fossil ichnites, constituting one of the most important paleoichnological sites in the world [37]. Dillehay [6] registered the existence of human footprints in the locality of Monte Verde located nearby Pilauco in the vicinity of the city of Puerto Montt, Chile. The find at Monte Verde is composed of one well preserved human footprint and two probable footprints, associated with remains of human occupations like a hearth and workshops [2,6]. Although the archaeological context has solid evidence of a steady human occupation of the site, so far this fossil footprint has not received a formal ichnotaxonomic description.

### Trace preservation and age

The infilling material had a notably different consistency compared to the more compacted trackbed. The infilling sediments have a slightly higher organic component and finer grain composition (S1 File). The information obtained from radiocarbon dating confirms that infill sediments were rapidly deposited and mild sediment mixing is present (Table 1). The trace does not present overtracks, which would have left progressively concave layers on top of the imprint. Consequently, it was most likely preserved during a fast-track burial event [48,49].

Various sedimentary environments allow the preservation of footprints including a proximity to water bodies, associated fine grained substrate with high viscosity due to increased water and organic contents, and the possibility of a rapid burial [48–50]. Peat deposits are apparently ideal environments, as demonstrated by the large number of tracksites found in this type of environment [38,50–53]. Microbial matts are easily developed under such conditions, facilitating a cleaner foot/sediment detachment, reducing the vacuum produced when the foot is lifted up and set away from the substrate [38,49]. Our trackway-making experiments produced footprint morphologies that are remarkably similar to previously analyzed human ichnites within thick microbial matts (>3 mm) [49].

Footprint morphology depends on three factors: sediment composition, foot shape and foot kinematics [51,52]. It is important to know the influence of these factors in order to obtain a clear picture of the conditions of footprint formation, especially when it is preserved deep into the sediment [53–56].

The experiments presented here demonstrate that a distally elongated mark of the hallux is particularly characteristic for a substrate saturated in water (Fig 4). This morphology has also been observed in experimental beach footprints in which *“…deeper footprints are characterized by a greater forefoot depth*, *with emphasis on the toe*, *than shallow footprints*.” [57]. The detailed digit morphology, particularly of the lateral digits, can be easily obliterated by mud collapses [23,46,58–60]. Sediment lumps can drop from the dorsal surface of the toes when the foot is lifted up over the sediment (Fig 5). These lumps might drop in front or fall inside the footprint depending on the magnitude of the forward movement of the trackmaker’s foot, explaining the unusual conical structure present inside the trace. This information allows us to infer that Pilauco’s ichnite might well correspond to a barefooted human print. It was made on a water-saturated substrate, in which the lateral digital impressions were obliterated by sediment collapse, the hallux left a distal elongated mark, and a sediment lump dropped from the trackmaker’s foot (Fig 5). The negative volume of the hallux mark and the sediment lump are similar (5 *versus* 7 cm^3^, respectively). The trace was likely buried fast, preserving its morphology. The measured depth of the Pilauco ichnite might have been compressed by more than 3 meters of overlying sediment, reducing its displacement rim.

Ages obtained for the Pilauco footprint asociated deposits are *circa* 15,600 cal yr BP (Table 1), and are comparable to the ones obtained for the Monte Verde II site [2]. The timing of the footprint is within a 1400 cal yr BP period, between the underlying youngest age in PB-6 and the oldest age of the PB-7 trackbed. Samples and their duplicates shows some deposit mixing (age difference of about 400 yr). However, the complete sequence remains strong and fearly logical (Table 1). Hence, we can confirm that this deposit has been mostly undisturbed, and casts no doubt about its antiquity.

Early human occupation in southern South America (Patagonia) has been the focus of intense debate over the recent years. Current detailed chronologies show that human presence in the area can be traced back as far as ∼15 kyr [2,10] with a period of ∼3500 years of coexistence with extinct megafauna. This suggests a complex dynamic between climatic and human-made environmental changes, occurring coevally at the end of the Pleistocene [61,62]. The human trace finding in Pilauco, ichnologically characterised as *Hominipes modernus*, adds a new and independent line of evidence on the colonisation of northern Patagonia, as has been continuously defended for more than 40 years by now based on scientific findings from the neighbouring Monte Verde site.

## Supporting information

S1 File Supporting information on the associated materia (lithics and fossils), taxonomic comparisons and additional experimental data. (DOCX)

S1 Fig Unconformity between coarse gravel (PB6) and sandy peat (base of PB7), grid AC10, the red arrow indicates the north. The stratigraphic context is the same in the grid AD14.
(TIFF)

S2 Fig Spatial distribution of the human footprint (ichnite), lithic material and gomphotere bones in grids 14AD, 14AC, 15AD and 15AC, in the northwestern side of Pilauco site. Numbers refers to the lithics presented in S1 Table.
(TIF)

S3 Fig Artifact 14AD-P173B-220111 made on aphanitic basalt. Primary flake with a distal point.
(TIF)

S4 Fig Flake 15AC-P185-27111 made on dacitic glass, bifacial knapping. (TIF)

S5 Fig Artifact 15AD-P126-2501 made on aphanitic basalt by tertiary reduction, with distal and lateral retouching. Probably a scraper.
(TIF)

S6 Fig Artifact. 14AA-P33-180213 made on aphanitic basalt. Flake with active distal and lateral links.
(TIF)

S7 Fig Artifact 17AA-P056-050213, made on dacitic glass. Primary flake with active distal and right lateral. Scraper.
(TIF)

S8 Fig Artifact 17AA-P81-120115, made on aphanitic basalt by tertiary reduction, with retouching. Scraper.
(TIF)

S9 Fig Image analysis of the anterior haft of the fossil ichnite. Keys were placed to facilitate the identification of features and their respective locations. A) X-Ray image showing the absence of clast (C) imbedded into the sediment lump (SL). Clasts are observed elsewhere under the ichnite (depth is not known), as well as 4 screws (s) used to build the base of the wooden structure that holds the entire sediment block. B) Picture of the ichnite surface as it was placed for X-Ray imaging. Note that desiccation cracks (d) were developed along the right side of the SL. Black arrow points out to the top of the hallux mark in both A and B images.
(TIFF)

S10 Fig Schematic view of the experimental setting. Trackmakers walked on the rehidrated fossilbed sediment.
(TIF)

S11 Fig Schematic representation of the sediment textural composition. Notice that trackbed samples have a slightly higher mud and gravel content than the rest. Infilling sediment has a lower gravel content.
(TIF)

S1 Table Petrographic and spatial position of artifacts, flakes and debitage in grids 14AC, 14AD, 15AD and 15AC in the northwestern side of Pilauco site. Numbers referred at S2 Fig.
(DOCX)
