# Supplementary material for: A late Pleistocene human footprint from the Pilauco archaeological site, northern Patagonia, Chile
Source: PLoS One. 2019 Apr 24;14(4):e0213572. doi: 10.1371/journal.pone.0213572 (PMC6481816; doi:10.1371/journal.pone.0213572)
Supplement: S1 File — (DOCX) [file pone.0213572.s001.docx]

**A late Pleistocene human footprint from the Pilauco Site, Chilean Patagonia**

Karen Moreno, Juan Enrique Bostelmann, Cintia Macías, Ximena Navarro-Harris, Ricardo De Pol-Holz, Mario Pino

**Supplementary Materials**

**1.-Lithics associated to the footprint.**

Among the material registered, there are about 80 lithic artefacts recovered from the occidental area of the site. Correspond to unstandardized flakes, characterized by unifacial marginal trimming, which would be used for scraping and cutting. These lithic tools were made from medium and fine basalt (80%) and quartzite (3%). Dacitic and rhyodacitic glass (17%) were knapped on foraneous rocks. 75% of the lithic tool collection is constituted by debitage.

In the 14AD grid at same level near the human ichnite, were registered artefacts, flakes and debitage. A total of 8 aphanite basalt and dacitic glass pieces were recovered *in situ* or over the screen. The debitage should have been produced by the knapping or as the product of reactivation of active edges by the use over other raw materials.

Other archaeological materials were registered in grids 14AC, 15AD and 15AC. The Fig S 2 shows the spatial relationships between the ichnite, the gomphotere bones and the lithic artefacts. The Table S.1 indicate the characteristics of the lithic artefacts, and the Figs. S3 to S5 include some examples of artefacts and flakes. In the grid 15AD was found one of the most elaborate artefacts of the entire Pilauco lithic collection (15AD-P126-250111, Fig. S5). The lithic technology is of an expeditive type. This means that it made in short time and with little effort to obtain the material. These are generaly local, such as basalt, and devitage was quickly discarded in-situ. This industry is known as “unifacial edge-trimmed flake tools” (Gruhn and Bryan, 2003; Dillehay et al., 2012).

**S1 Table**: Petrographic and spatial position of artefacts, flakes and debitage in grids 14AC, 14AD, 15AD and 15AC in the northwestern side of Pilauco site. Numbers referred to S2.

| **Artefact** | **Number*** | **Type** | **Lithology** | **North** | **East** | **Local Elevation** | **Quantity** | **Sup. Fig.** |
| --- | --- | --- | --- | --- | --- | --- | --- | --- |
| 14AD-P17B-22011 | 1 | flake | aphanitic basalt | 92 | 92 | 330 | 1 | S3 |
| 14AD-P045-91210 | 2 | flake | dacitic glass | 26 | 58 | 366 | 1 |  |
| 14AD-P173-220111 | 3 | debitage | dacitic glass | 85 | 87 | 329 | 1 |  |
| 14AD-P152-180111 | 4 | debitage | dacitic glass | 19 | 38 | 336 | 1 |  |
| 14AD-P153-180111 | 5 | debitage | dacitic glass | 19 | 38 | 336 | 1 |  |
| 14AD-P154-180111 | 6 | debitage | dacitic glass | 50 | 50 | 336 | 1 |  |
| P155-180111-14AD | 7 | debitage | dacitic glass | 46 | 96 | 357 | 3 |  |
| 14AD-P030-041012 | 8 | debitage | dacitic glass | 50 | 50 | 380 | 2 |  |
| 15AD-P126-250111 | 9 | artefact | aphanitic basalt | 88 | 22 | 361 | 1 | S4 |
| 15AD-P048-101210 | 10 | debitage | dacitic glass | 50 | 50 | 383 | 2 |  |
| 15AC-P185-270111 | 11 | flake | dacitic glass | 50 | 50 | 365 | 1 | S5 |
| 15AC-P186-270111 | 12 | debitage | dacitic glass | 50 | 50 | 365 | 1 |  |
| 15AC-P045-161210 | 13 | debitage | dacitic glass | 38 | 34 | 302 | 2 |  |

**2.- Figure captions of the section: “Lithics associated to the footprint”**

**S1 Fig.** Unconformity between coarse gravel (PB6) and sandy peat (base of PB7), grid AC10, the red arrow indicates the north. The stratigraphic context is the same in the grid AD14.

**S2 Fig.** Spatial distribution of the human footprint (ichnite), lithic material and gomphotere bones in grids 14AD, 14AC, 15AD and 15AC, in the northwestern side of Pilauco site. Numbers refers to the lithics presented in Table S1.

**S3 Fig**: Artefact 14AD-P173B-220111 made on aphanitic basalt. Primary flake with a distal point.

**S4 Fig.** Flake 15AC-P185-27111 made on dacitic glass, bifacial knapping.

**S5 Fig.** Artefact 15AD-P126-2501 made on aphanitic basalt by tertiary reduction, with distal and lateral retouching. Probably a scraper.

**S6 Fig.** Artifact. 14AA-P33-180213 made on aphanitic basalt. Flake with

active distal and lateral links.

**S7 Fig.** Artifact 17AA-P056-050213, made on dacitic glass. Primary flake with active distal and right lateral. Scraper.

**S8 Fig.** Artifact 17AA-P81-120115, made on aphanitic basalt by tertiary reduction, with retouching. Scraper.

**3.-Palaeontological assemblage**

The extinct faunal remains exhumed at Pilauco include xenarthran dermal bones currently assigned to giant ground sloths; large skeletal elements of Proboscidea: *Notiomastodon* *platensis*; Perissodactyla: *Equus andium*; Cetartiodactyla: *Hemiauchenia paradoxa* and *Pudu* sp*.*; Carnivora: *Conepatus* sp.; and Rodentia: *Loxodontomys micropus*, *Myocastor coypus*. Coleopterans; unidentified hair; and large coprolites containing equid includyng ruminant parasites. Marks on *Notiomastodon platensis* bones could also suggest the presence of large felids scavenging the carcases [1-8].

Plant materials include leaves, seeds and pollen typical of North Patagonic open forest vegetation (unlike the actually present Valdivian forest) such as *Prumnopitys andina*, *Podocarpus nubigenus* and *Saxegothaea conspicua*, Fitzroya/Pilgerodendron, Myrtaceae, *Maytenus sp.,* *Aextoxicon punctatum* and *Nothofagus* *dombeyi* type, as well as aquatic vegetation [9,10].

**4.- Figure captions of the section: “Systematic Paleoichnology of the Pilauco Trace”**

**S9 Fig.** Image analysis of the anterior haft of the fossil ichnite. Keys were placed to facilitate the identification of features and their respective locations. A) X-Ray image showing the absence of clast (C) imbedded into the sediment lump (SL). Clasts are observed elsewhere under the ichnite (depth is not known), as well as 4 screws (s) used to build the base of the wooden structure that holds the entire sediment block. B) Picture of the ichnite surface as it was placed for X-Ray imaging. Note that desiccation cracks (d) were developed along the right side of the SL. Black arrow points out to the top of the hallux mark in both A and B images.

**5.- Figure captions for the experimental trackways**

**S10 Fig.** Schematic view of the experimental setting. Trackmakers walked on the rehidrated fossilbed sediment.

**S11 Fig.** Schematic representation of the sediment textural composition. Notice that trackbed samples have a slightly higher mud and gravel content than the rest. Infilling sediment has a lower gravel content.

**6- Ichnological comparisons to ground sloths footprints**

Ground sloth fossil footprints and tracks has been reported in the scientific literature since the end of the 19th century [11-14], but ichnotaxonomic treatment for them was only achieved during the late decades of the past century. The Argentinean paleontologist Rodolfo Casamiquela was the first one to use an ichnological nomenclature for late Miocene to Plio-Pleistocene ground sloths footprints of south central Argentina [15,16]. Since those pioneering works, vertebrate ichnologists have reach important advances in the recognition and taxonomic characterization of them.

Presently, five monotypic ichnospecies are referred to fossil ground sloth footprints and tracks: *Megatherichnum oportoi* Casamiquela 1974 [15], *Acugnaichnus dorregoensis* Casamiquela 1983 [16], *Iribarichnum megamericanum* Casamiquela 1983[16], *Mylodontidichnum rosalensis* Aramayo and Manera de Bianco 1987[17], and *Neomegatherichnum pehuencoensis* Aramayo and Manera de Bianco, 1987 [17, 18]. In spite of this diversity, only the genus *Megatherichnum*, *Mylodontidichnum* and *Neomegatherichnum* have being reviewed following modern ichnological criteria, while *Iribarichnum megamericanum* is considered a *nomen nudum* [19].

Ground sloth fossil footprints are usually large, over 50 cm to 100 cm long, circular to elliptical shape and are preserved as a concave epirelief. They frequently display thick and elevated ridges at their anterior and lateral margins. Usually they lack plantar structures, and/or the marks of more than three digits. Manus impressions are circular to sub-circular while pes marks are usually sub-elliptical or kidney-shaped. They frequently show the presence of a large, single, inwardly or backwardly directed sub-triangular claw mark [18, 20]. *Hominipes modernus* from the Pilauco site clearly differs from all these ichnospecies by its small size, slender condition, straight plantar surface, anterior an lateral borders without massive ridges, anteriorly projected digits, presence of a rounded, well-delineated medial constriction (“ball”), and the absence of any type of backwardly projected claw mark.

**6.-References**

1. Canales-Brellenthin P (2008) El ratón de pié chico del Sitio Pilauco. In: Pino M, editor. Pilauco: Un sitio complejo del Pleistoceno tardío Osorno, Norpatagonia chilena. Valdivia, Chile: Universidad Austral de Chile - Imprenta América. pp. 77-80.

2. González EG, Prevosti FJ, Pino M (2010) Primer registro de mephitidae (carnivora: mammalia) para el pleistoceno de Chile. Magallania 38: 239-248.

3. Labarca R, Pino M, Recabarren OP (2013) Los Lamini (Cetartiodactyla: Camelidae) extintos del yacimiento de Pilauco (Norpatagonia chilena): aspectos taxonómicos y tafonómicos preliminares. Estudios Geológicos 69: 255-269.

4. Labarca R, Recabarren OP, Canales-Brellenthin P, Pino M (2014) The gomphotheres (Proboscidea: Gomphotheriidae) from Pilauco site: Scavenging evidence in the Late Pleistocene of the Chilean Patagonia. Quaternary International 352: 75-84.

5. Pino M, Chávez-Hoffmeister M, Navarro-Harris X, Labarca R (2013) The late Pleistocene Pilauco site, Osorno, south-central Chile. Quaternary International 299: 3-12.

6. Recabarren OP, Pino M, Alberdi MT (2014) La Familia Gomphotheriidae en América del Sur: Evidencia de molares al norte de la Patagonia chilena. Journal of Vertebrate Paleontology 70: e001.

7. Recabarren OP, Pino M, Cid I (2011) New record of Equus (Mammalia: Equidae) from the late Plaistocene of central-south Chile. Revista Chilena de Historia Natural 84: 535-542.

8. Recabarren OP, Buckley M, García N, Pino M. Determinación de la secuencia de aminoácidos del colágeno (I) en restos de gonfoterio (Proboscidea, Gomphotheriidae), del Pleistoceno tardío del yacimiento Pilauco (sur de Chile). ; 2015. pp. 212-214.

9. Abarzúa A, Lobos V, Martel-Cea A (2016) ¡Pequeño testigo de grandes cambios!: polen, semillas y carbones escondidos en Pilauco. In: Pino M, editor. El Sitio Pilauco Osorno, Patagonia Noroccidental de Chile. Valdivia, Chile: Universidad Austral de Chile - Imprenta América. pp. 112-127.

10. Jarpa L (2008) Las diatomeas del sitio Pilauco. In: Pino M, editor. Pilauco: Un sitio complejo del Pleistoceno tardío Osorno, Norpatagonia chilena. Valdivia, Chile: Universidad Austral de Chile - Imprenta América. pp. 43-48.

11. Cope ED (1883) The Nevada biped tracks. American Naturalist 17(1): 69-71.

12. Marsh OC (1883). On the supposed human footprints recently found in Nevada. American Journal of Science 3(26): 139-140.

13. Blake WP (1884). The Carson-City ichnolites. Science 4 (85): 273–276.

14. Stock Ch. (1936) Sloth tracks in the Carson prison. Westways 28: 26-27.

15. Casamiquela RM (1974). El bipedismo de los megaterioideos. Estudio de pisadas fósiles en la Formación Río Negro típica. Ameghiniana 11: 249–282.

16. Casamiquela RM (1983). Pisadas del Pleistoceno (¿Superior?) del Balneario de Monte Hermoso, Buenos Aires — La confirmación del andar bipedal en los megaterioideos. Cuadernos del Instituto Superior “Juan XXIII”: 1–21.

17. Aramayo SA, Manera de Bianco T (1987) Hallazgo de una icnofauna continental (Pleistocene tardío) en la localidad de Pehuen Có, Provincia de Buenos Aires, Argentina. Parte I: Edentata, Litopterna, Proboscidea. Parte II: Carnivora, Artiodactyla y Aves. 4° Congreso Latinoamericano de Geología, Actas, Santa Cruz de la Sierra, Bolivia: 516–547.

18. Melchor RN, Pérez M, Cardonatto MC, Umazano AM (2015) Late Miocene ground sloth footprints and their paleoenvironment: Megatherichnum oportoi revisited. Palaeogeography Palaeoclimatology Palaeoecology: 439: 126–143.

19. Aramayo SA, Manera de Bianco T, Bastianelli NV, Melchor RN (2015) Pehuen Co: Updated taxonomic review of a late Pleistocene ichnological site in Argentina. Palaeogeography, Palaeoclimatology, Palaeoecology 439: 144–165.

20. McDonald HG (2007) Biomechanical inferences of locomotion in ground sloths: integrating morphological and track data. Museum of Natatural History Science. Bulletin 42: 201–208.
