# Supplementary figures and images for: A late Pleistocene human footprint from the Pilauco archaeological site, northern Patagonia, Chile
Source: PLoS One. 2019 Apr 24;14(4):e0213572. doi: 10.1371/journal.pone.0213572 (PMC6481816; doi:10.1371/journal.pone.0213572)

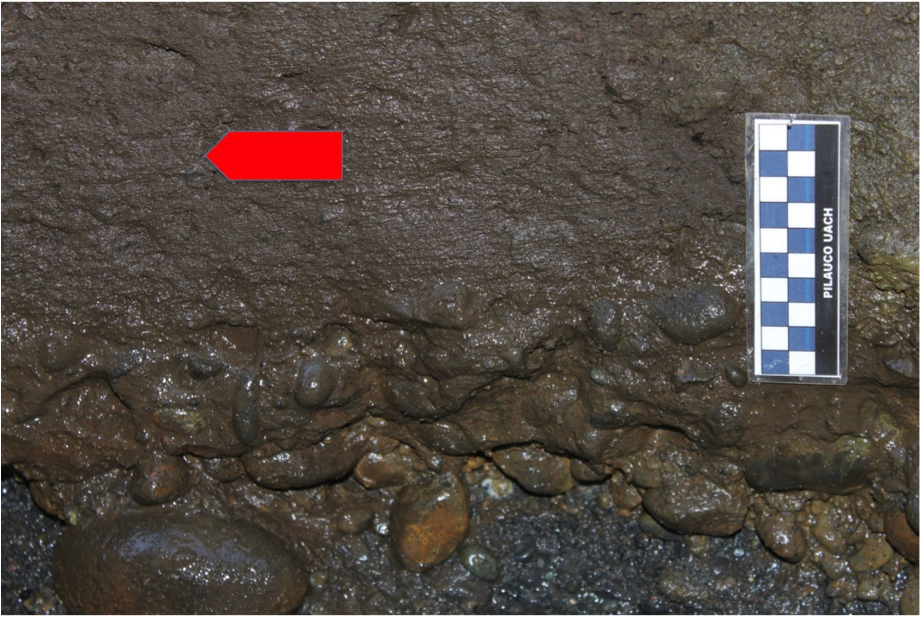

Supplement: S1 Fig — The stratigraphic context is the same in the grid AD14. (TIFF) [file pone.0213572.s002.tiff]

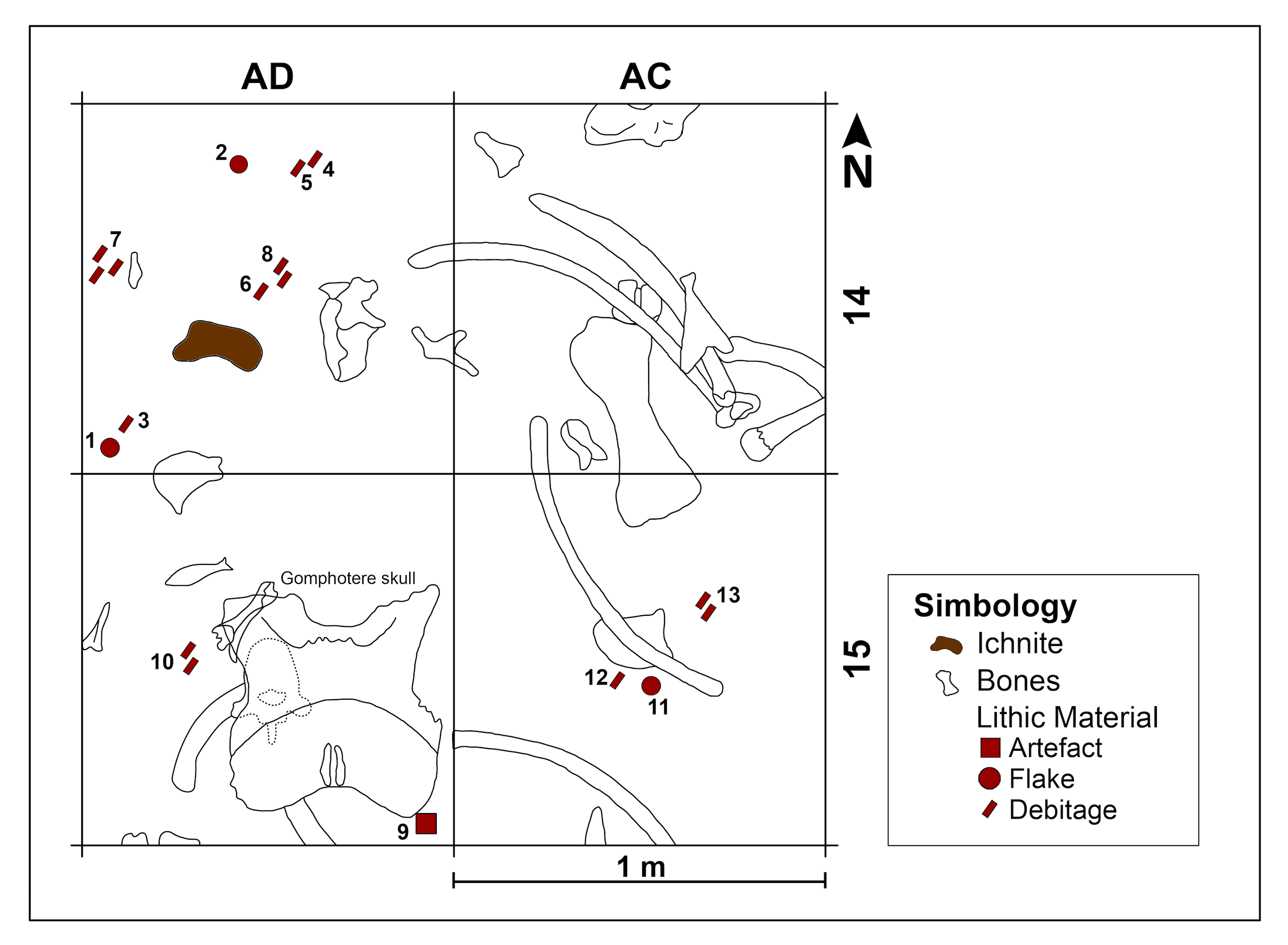

Supplement: S2 Fig — Numbers refers to the lithics presented in S1 Table. (TIF) [file pone.0213572.s003.tif]

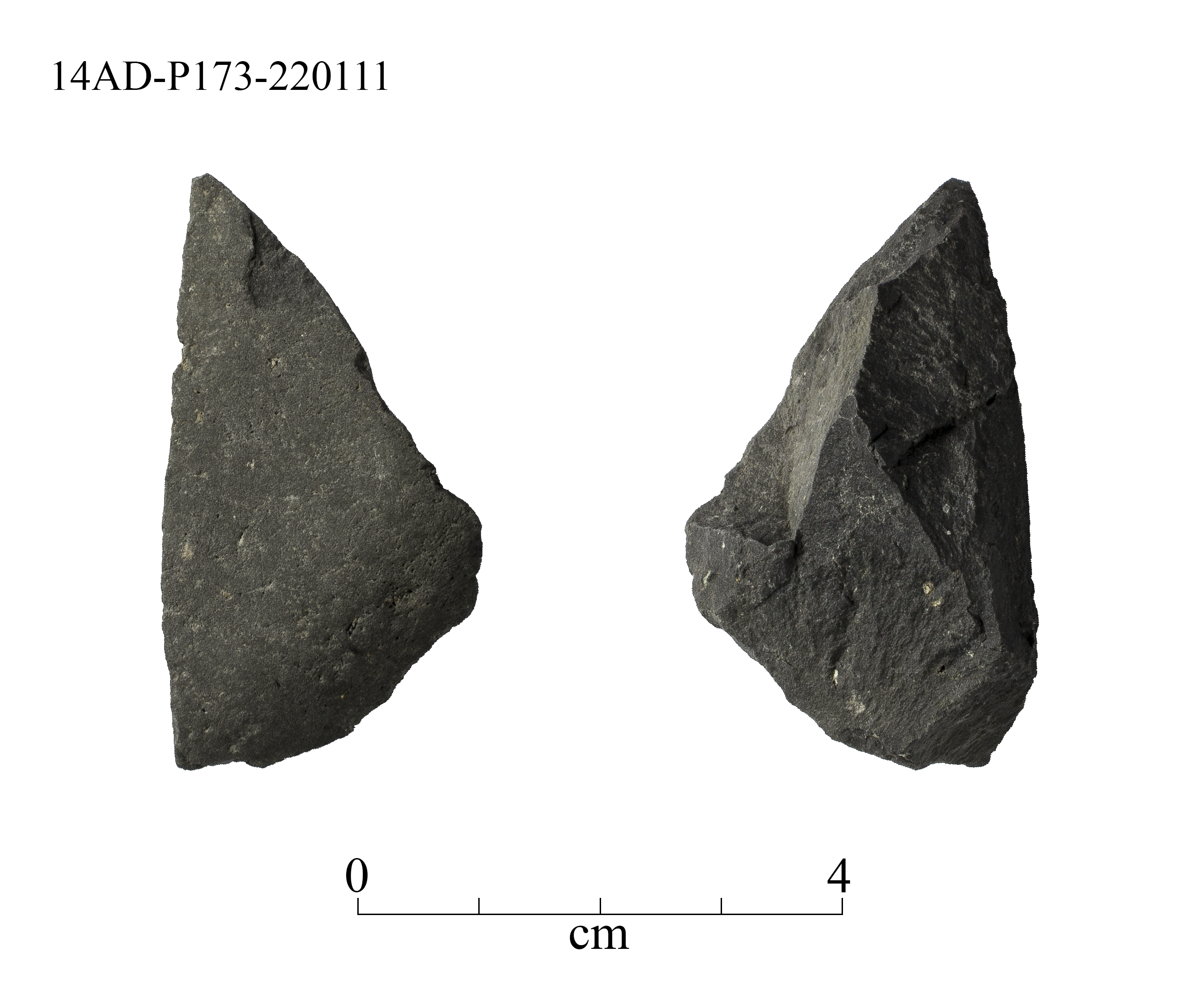

Supplement: S3 Fig — Primary flake with a distal point. (TIF) [file pone.0213572.s004.tif]

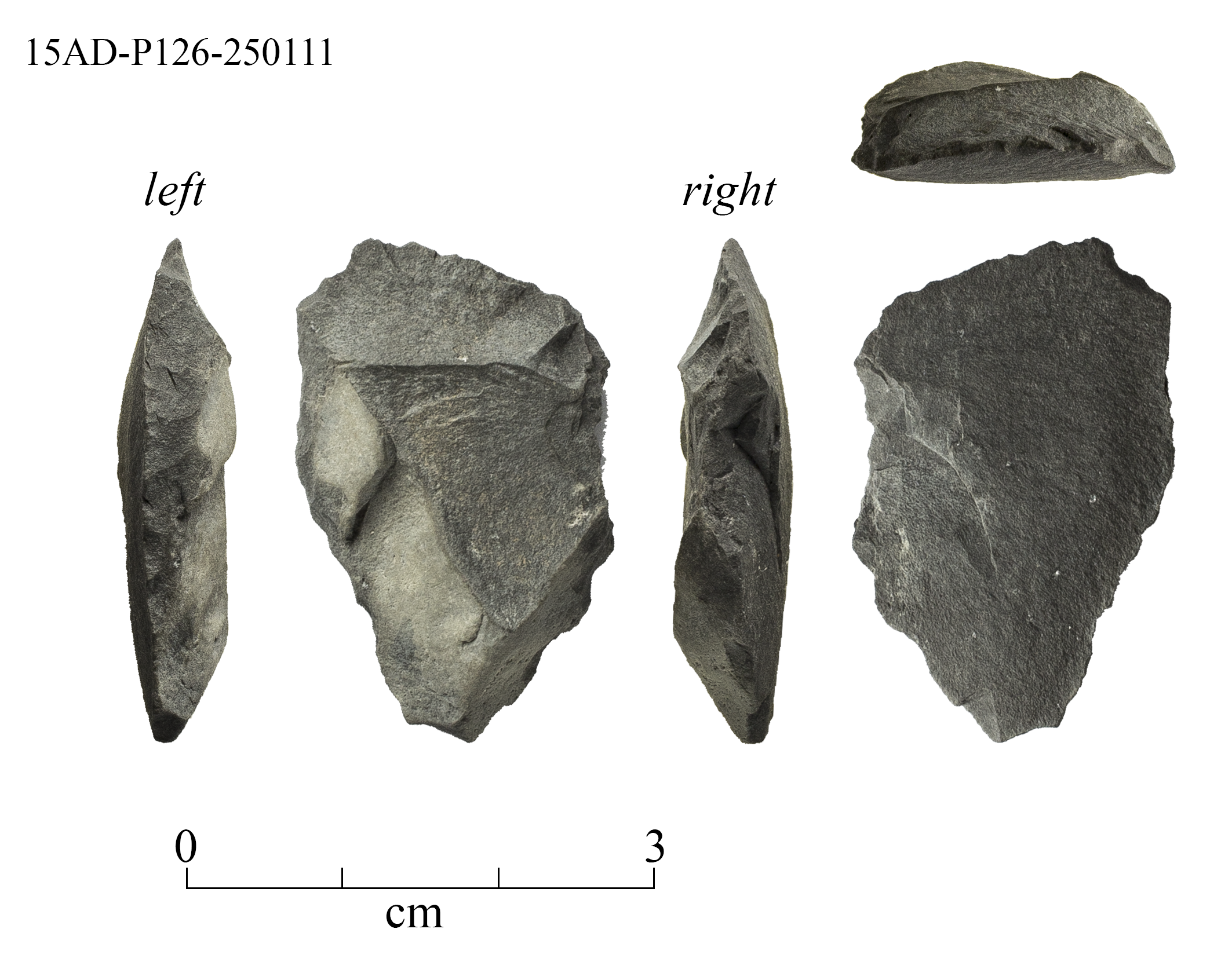

Supplement: S4 Fig — (TIF) [file pone.0213572.s005.tif]

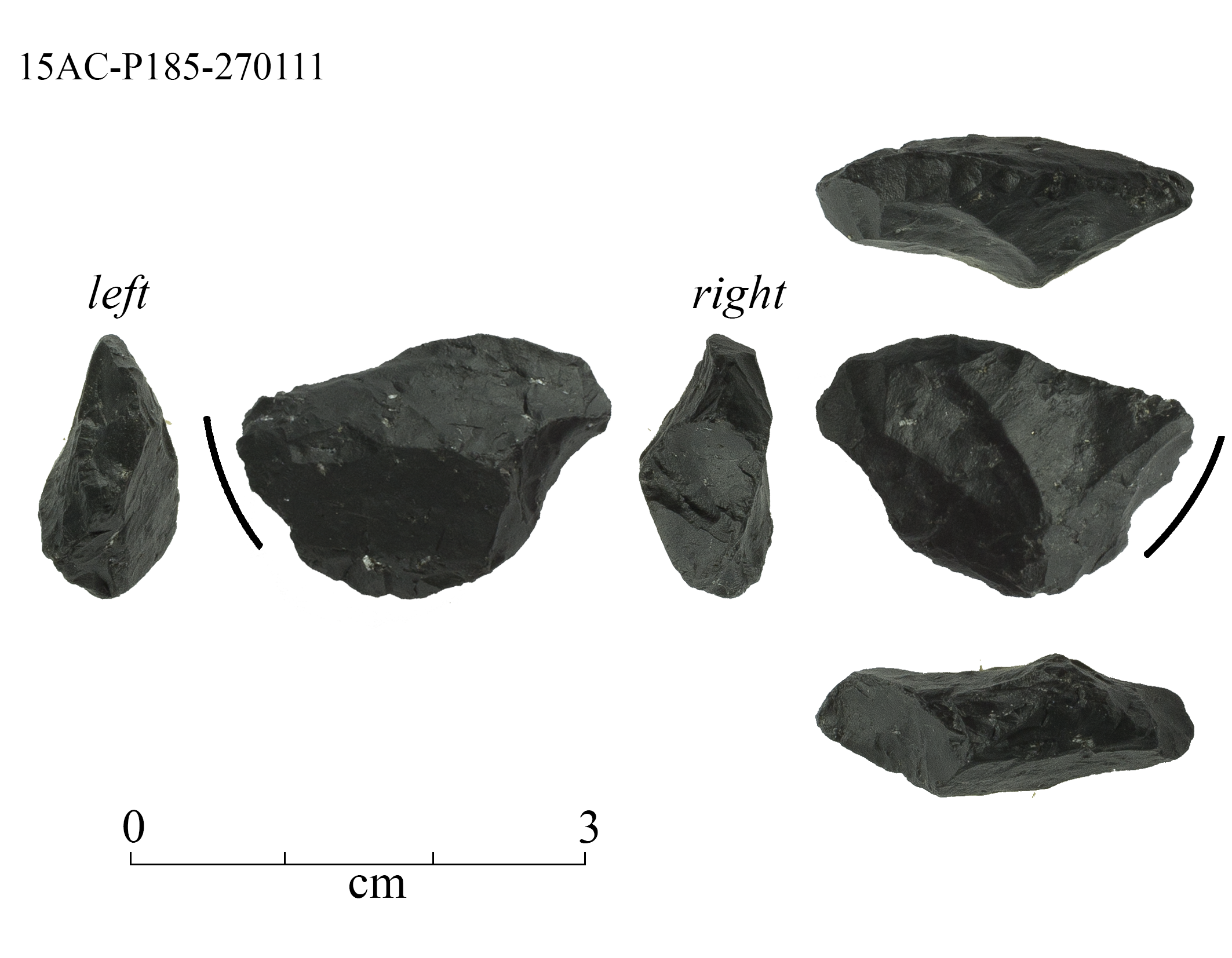

Supplement: S5 Fig — Probably a scraper. (TIF) [file pone.0213572.s006.tif]

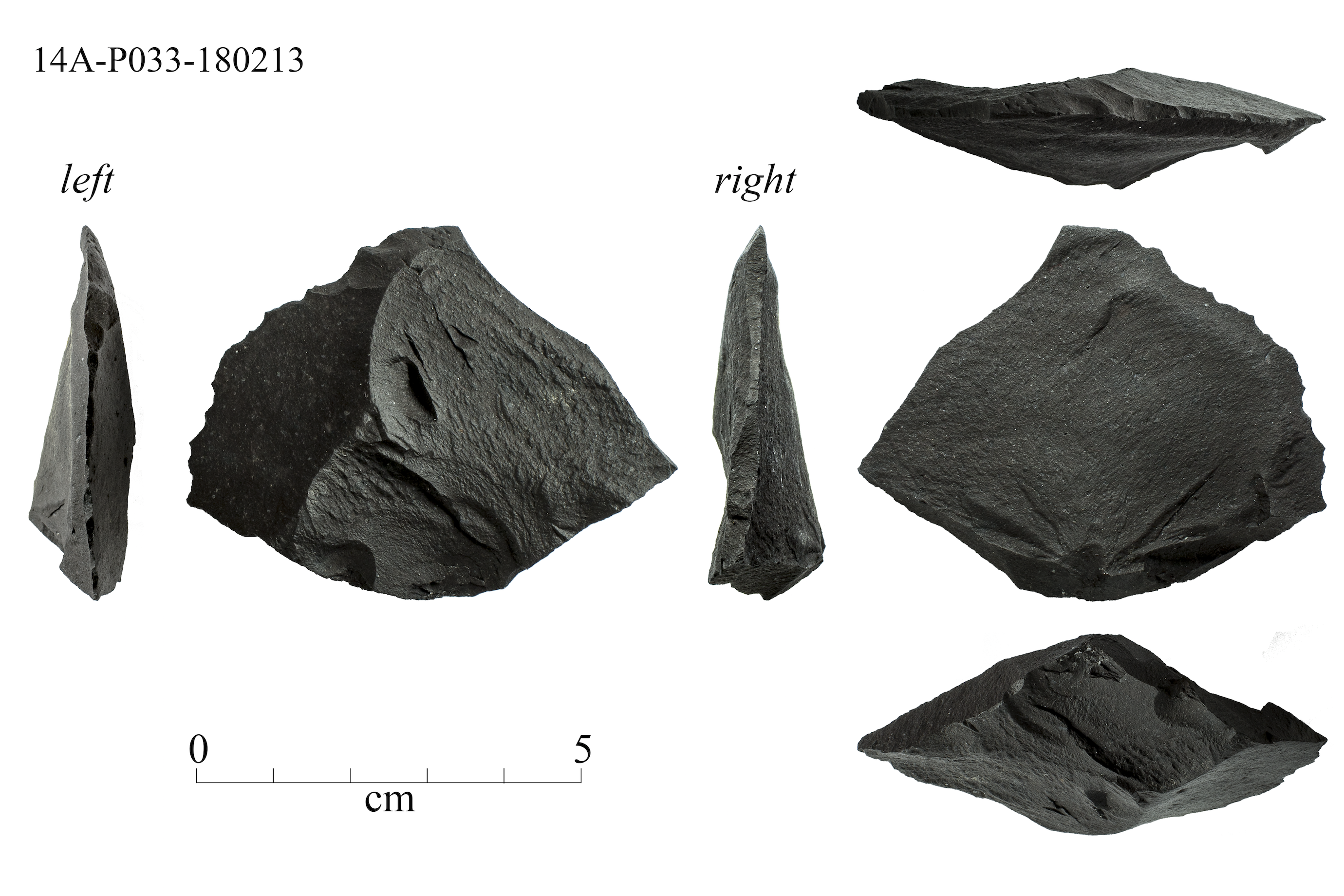

Supplement: S6 Fig — 14AA-P33-180213 made on aphanitic basalt. Flake with active distal and lateral links. (TIF) [file pone.0213572.s007.tif]

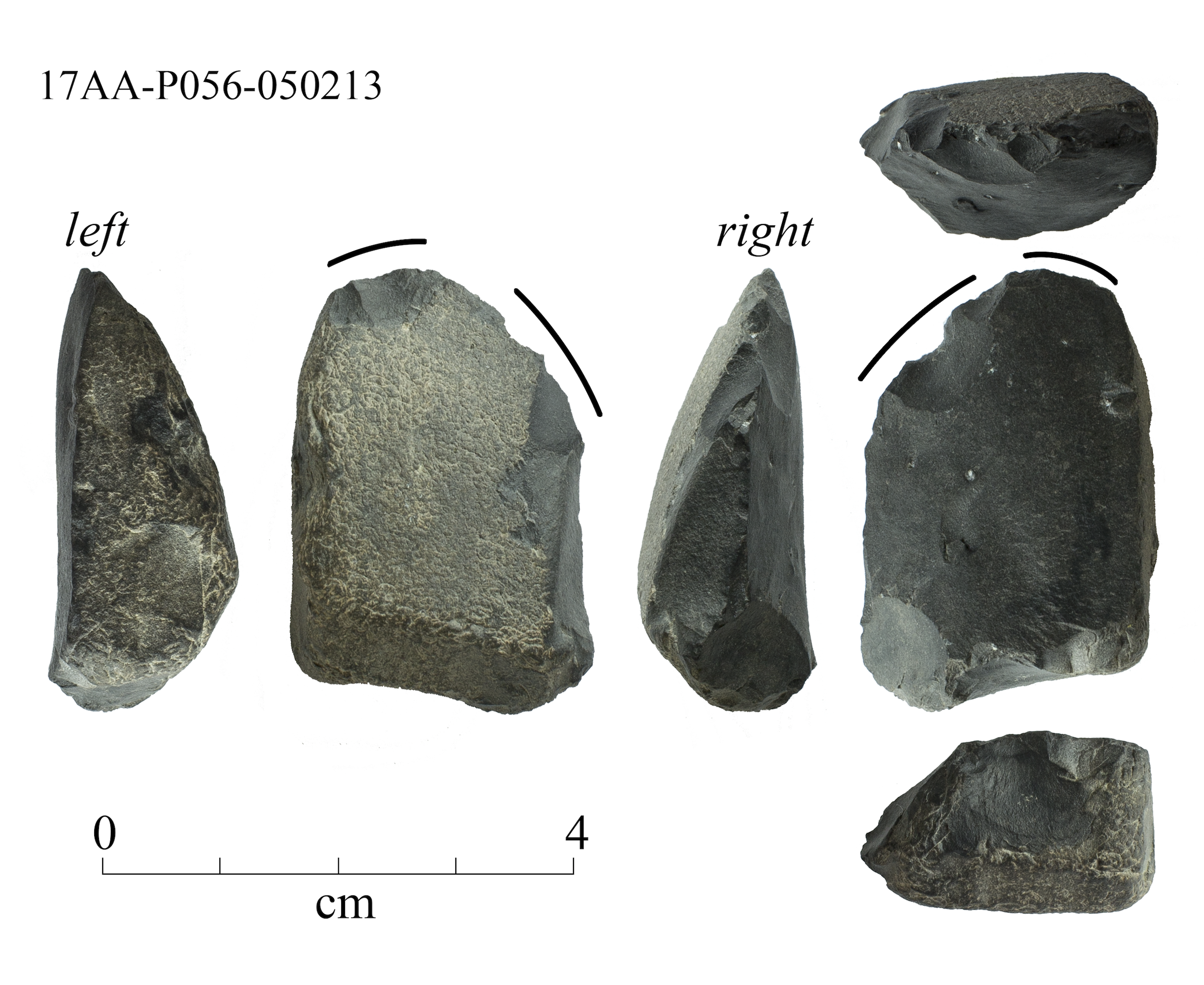

Supplement: S7 Fig — Primary flake with active distal and right lateral. Scraper. (TIF) [file pone.0213572.s008.tif]

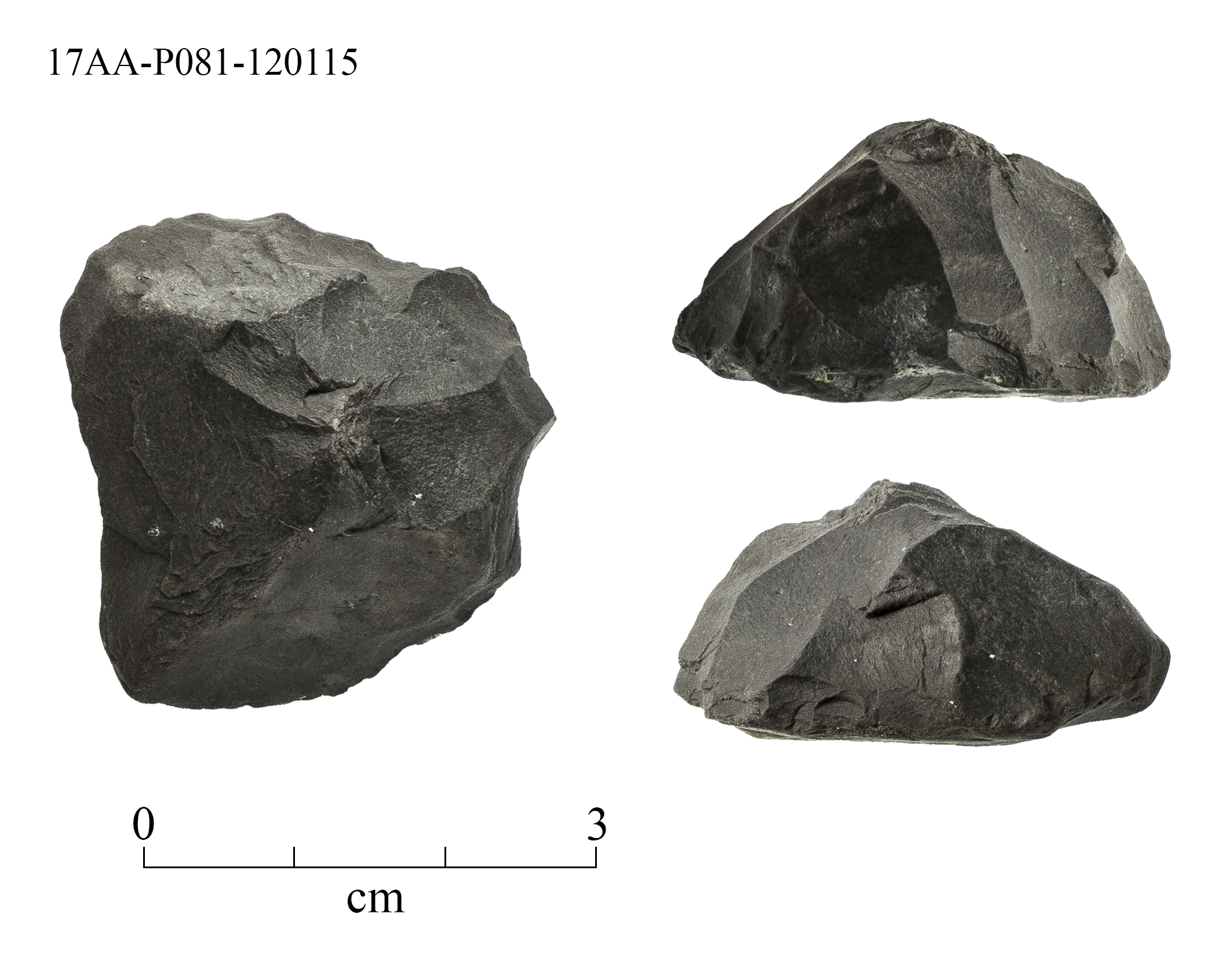

Supplement: S8 Fig — Scraper. (TIF) [file pone.0213572.s009.tif]

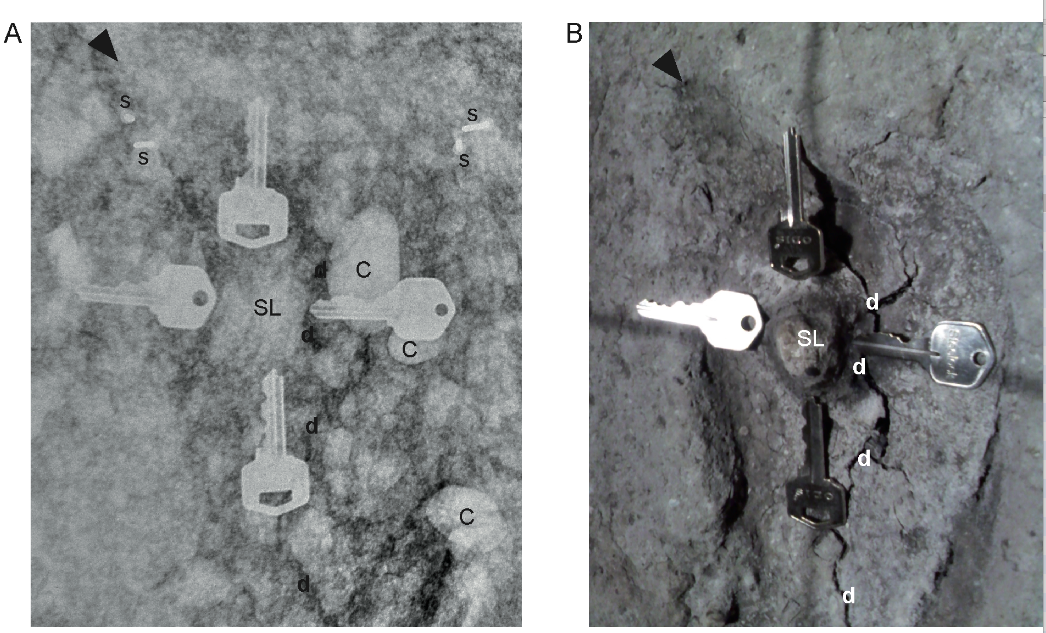

Supplement: S9 Fig — Keys were placed to facilitate the identification of features and their respective locations. A) X-Ray image showing the absence of clast (C) imbedded into the sediment lump (SL). Clasts are observed elsewhere under the ichnite (depth is not known), as well as 4 screws (s) used to build the base of the wooden structure that holds the entire sediment block. B) Picture of the ichnite surface as it was placed for X-Ray imaging. Note that desiccation cracks (d) were developed along the right side of the SL. Black arrow points out to the top of the hallux mark in both A and B images. (TIFF) [file pone.0213572.s010.tiff]

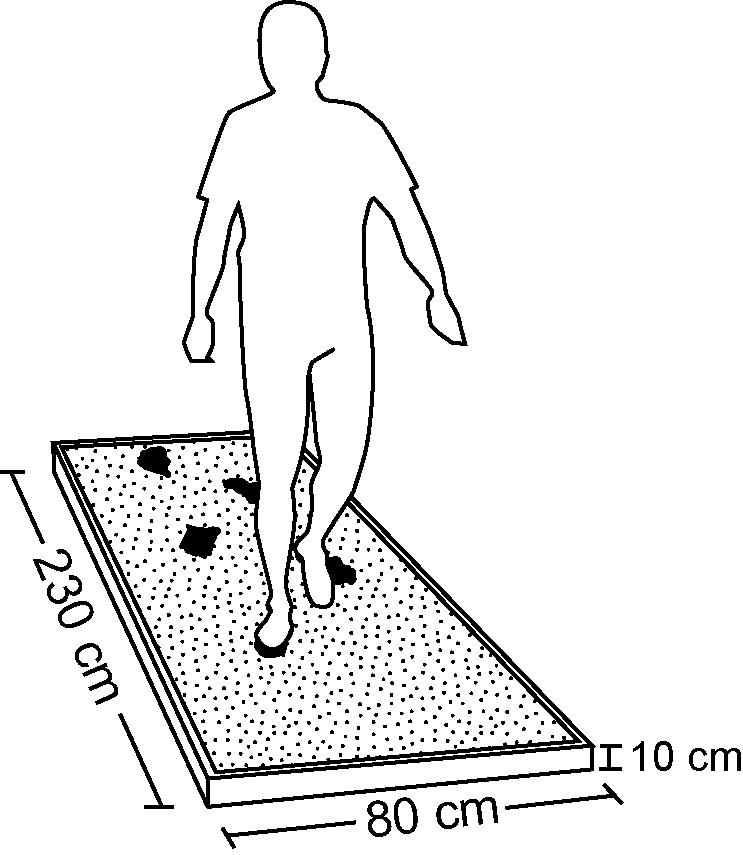

Supplement: S10 Fig — Trackmakers walked on the rehidrated fossilbed sediment. (TIF) [file pone.0213572.s011.tif]

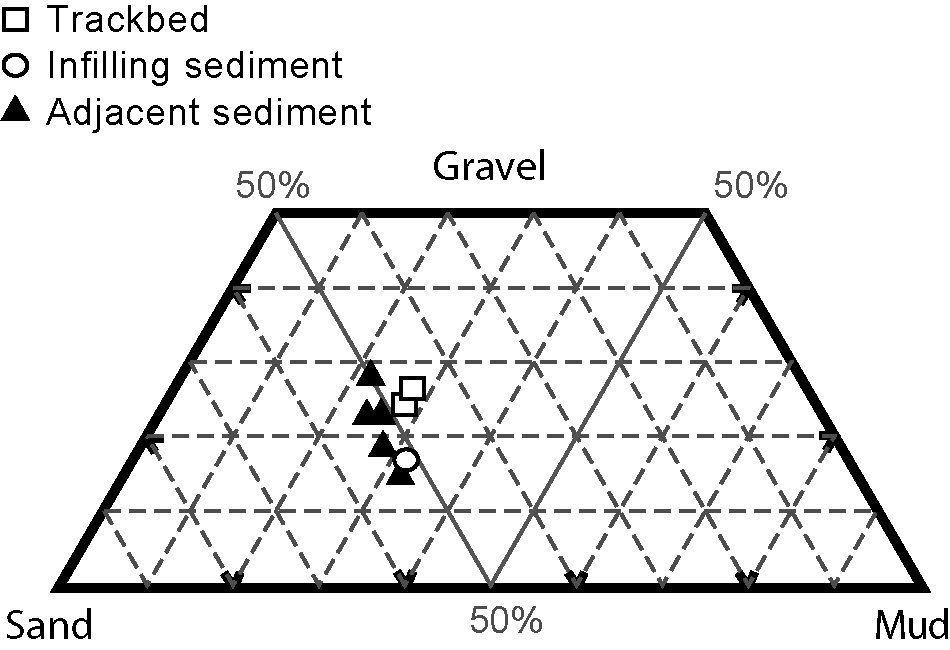

Supplement: S11 Fig — Notice that trackbed samples have a slightly higher mud and gravel content than the rest. Infilling sediment has a lower gravel content. (TIF) [file pone.0213572.s012.tif]
