# Supplementary material for: A late Pleistocene human footprint from the Pilauco archaeological site, northern Patagonia, Chile
Source: PLoS One. 2019 Apr 24;14(4):e0213572. doi: 10.1371/journal.pone.0213572 (PMC6481816; doi:10.1371/journal.pone.0213572)
Supplement: S1 Table — Numbers referred at S2 Fig. (DOCX) [file pone.0213572.s013.docx]

**S1 Table**: Petrographic and spatial position of artefacts, flakes and debitage in grids 14AC, 14AD, 15AD and 15AC in the northwestern side of Pilauco site. Numbers referred to S2.

| **Artefact** | **Number*** | **Type** | **Lithology** | **North** | **East** | **Local Elevation** | **Quantity** | **Sup. Fig.** |
| --- | --- | --- | --- | --- | --- | --- | --- | --- |
| 14AD-P17B-22011 | 1 | flake | aphanitic basalt | 92 | 92 | 330 | 1 | S3 |
| 14AD-P045-91210 | 2 | flake | dacitic glass | 26 | 58 | 366 | 1 |  |
| 14AD-P173-220111 | 3 | debitage | dacitic glass | 85 | 87 | 329 | 1 |  |
| 14AD-P152-180111 | 4 | debitage | dacitic glass | 19 | 38 | 336 | 1 |  |
| 14AD-P153-180111 | 5 | debitage | dacitic glass | 19 | 38 | 336 | 1 |  |
| 14AD-P154-180111 | 6 | debitage | dacitic glass | 50 | 50 | 336 | 1 |  |
| P155-180111-14AD | 7 | debitage | dacitic glass | 46 | 96 | 357 | 3 |  |
| 14AD-P030-041012 | 8 | debitage | dacitic glass | 50 | 50 | 380 | 2 |  |
| 15AD-P126-250111 | 9 | artefact | aphanitic basalt | 88 | 22 | 361 | 1 | S4 |
| 15AD-P048-101210 | 10 | debitage | dacitic glass | 50 | 50 | 383 | 2 |  |
| 15AC-P185-270111 | 11 | flake | dacitic glass | 50 | 50 | 365 | 1 | S5 |
| 15AC-P186-270111 | 12 | debitage | dacitic glass | 50 | 50 | 365 | 1 |  |
| 15AC-P045-161210 | 13 | debitage | dacitic glass | 38 | 34 | 302 | 2 |  |
